# Stability Evaluation of DMT and Harmala Alkaloids in Ayahuasca Tea Samples

**DOI:** 10.3390/molecules25092072

**Published:** 2020-04-29

**Authors:** Gabriela de Oliveira Silveira, Rafael Guimarães dos Santos, Felipe Rebello Lourenço, Giordano Novak Rossi, Jaime E. C. Hallak, Mauricio Yonamine

**Affiliations:** 1 Department of Clinical and Toxicological Analyses, School of Pharmaceutical Sciences, University of São Paulo, São Paulo 05508-000, Brazil; yonamine@usp.br; 2 Department of Neurosciences and Behaviour, University of São Paulo, Ribeirão Preto 14049-900, Brazil; banisteria@gmail.com (R.G.d.S.); gionorossi@gmail.com (G.N.R.); jechallak@gmail.com (J.E.C.H.); 3 National Institute of Science and Technology—Translational Medicine, Ribeirão Preto 14049-900, Brazil; 4 Department of Pharmacy, School of Pharmaceutical Sciences, University of São Paulo, São Paulo 05508-000, Brazil; feliperl@usp.br

**Keywords:** ayahuasca, stability, harmala alkaloids, DMT, LC-MS/MS

## Abstract

Ayahuasca tea is a hallucinogenic beverage used for religious purposes in Brazil and many other countries that has therapeutic potential in the treatment of some mental health disorders. In the context of psychedelic research, quantification of the tea’s main alkaloids prior to its administration in animal or human studies is essential. For this reason, this study aims to provide information regarding the stability of the main ayahuasca alkaloids (dimethyltryptamine, DMT; harmine, HRM; tetrahydroharmine, THH; harmaline, HRL) in three different conditions: (1) A year stored in a refrigerator either in plastic or glass containers, (2) seven days at 37 °C to reproduce usual mail transportation, and (3) after three freeze–thaw cycles. Samples were quantified after a dilute-and-shoot procedure using liquid chromatography tandem mass spectrometry (LC-ESI-MS/MS). There was no significant degradation of DMT concentration over time in all tested conditions. Harmala alkaloids (THH, HRL, and HRM) showed important variations after long-term and high-temperature storages. Although DMT has proven to be stable in all studied conditions, the harmala alkaloids revealed intense degradation and even concentration increment. This may be caused by degradation, alkaloid inter-conversion, and leaching from tea precipitate material. Therefore, ayahuasca quantification before administration in controlled sets is mandatory.

## 1. Introduction

**Figure 1:**
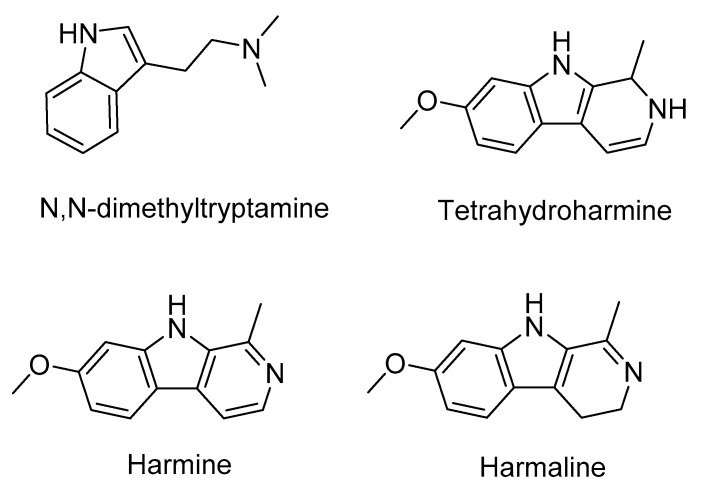
Chemical structures of the main ayahuasca alkaloids.

Ayahuasca tea is an indigenous hallucinogenic/psychedelic beverage prepared by the decoction of Amazonian plants containing dimethyltryptamine (DMT) and β-carboline or harmala alkaloids (harmine, harmaline, and tetrahydroharmine), Figure 1 [1,2,3]. The use of ayahuasca for shamanic and spiritual purposes dates from the pre-Columbian period in the Amazon basin region [4]. Currently, ayahuasca is used by various religious groups widespread in Brazil, North America, and Europe and is usually prepared by the decoction of the *Psychotria viridis* and *Banisteriopsis caapi* plants [3,5,6,7]. The synergistic effect that occurs between the β-carboline alkaloids (monoamine oxidase type A inhibitors) present in the *B. caapi* vine and the hallucinogen DMT present in the leaves of *P. viridis* allows for the final hallucinogenic response in the central serotoninergic receptors [7,8].

Because of its unique characteristics, the interest in studying ayahuasca is continuously growing. More recently, concerning the global trend in psychedelic therapy research, the therapeutic potential of this plant preparation has been studied as an alternative in the treatment of addiction [9,10,11], depression [12,13,14,15,16], and other mental health disorders [17,18]. In this context, the quantification of ayahuasca’s main alkaloids prior to its administration in animal or human studies is essential, as well as the knowledge of the tea’s sample origin, storage, and transportation conditions. Various analytical methods have been reported in the literature for the analysis of ayahuasca’s botanical preparations; these include solid-phase extraction (SPE), solid-phase microextraction (SPME), liquid–liquid extraction (LLE), and dilute-and-shoot for sample preparation prior to liquid chromatography or gas chromatography, both coupled to mass spectrometry and other detector types [19,20,21,22,23,24,25,26].

It is known that religious and scientific groups may keep the beverage stored for over a year after decoction. Therefore, this short communication aims to provide more detailed information regarding ayahuasca alkaloids’ stability in three different conditions: (1) A year stored in a refrigerator either in a plastic or glass container (4–8 °C), (2) 7 days in a high-temperature environment that mimics usual mail transportation (37 °C), and (3) after three freeze–thaw cycles for the case of re-analysis.

## 2. Results

### 2.1. LC-ESI-MS/MS Analysis

**Figure 2:**
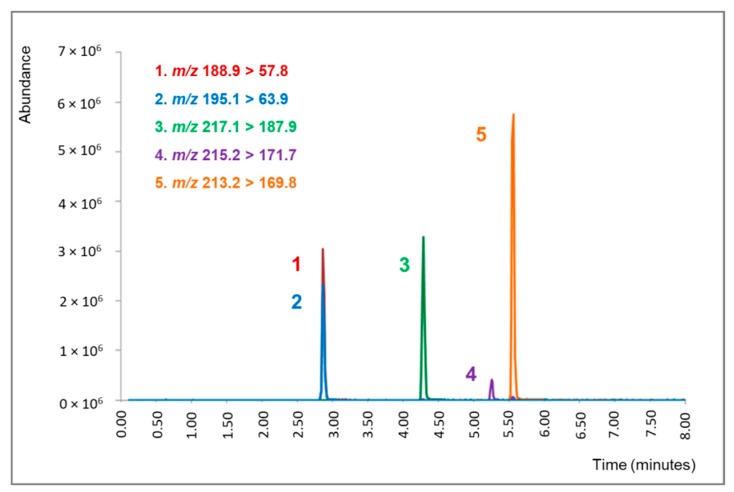
Chromatogram obtained from an ayahuasca sample containing 1. dimethyltryptamine (DMT, 223.0 ng/mL), 2. DMT-*d*_6_ (internal standard), 3. tetrahydroharmine (THH, 226.5 ng/mL), 4. harmaline (HRL, 45.1 ng/mL) and 5. harmine (HRM, 257.5 ng/mL) after the dilute-and-shoot procedure. The *m*/*z* shown above was used for the quantification of the analytes.

Figure 2 shows a chromatogram obtained from an ayahuasca tea sample containing DMT, harmine (HRM), harmaline (HRL), tetrahydroharmine (THH), and DMT-d_6_ (internal standard) after the dilute-and-shoot procedure using LC-ESI-MS/MS.

The method used for quantification was routinely conducted in our laboratory, and it was previously fully validated according to international guidelines [25]. Limits of detection and quantification were 1.0 and 1.5 ng/mL, respectively, for all analytes. Inter-assay precision, intra-assay precision, and accuracy were also assessed (three quality control levels, *n* = 6, 3 days), and their values remained within the acceptance criteria. The precision relative standard deviation (RSD) ranged from 5.4% (HRM, intra-assay, high-quality control (HQC)) to 15.4% (HRM, inter-assay, low-quality control (LQC)). Accuracy varied from 86.9% (HRM, medium quality control (MQC)) to 114.4% (HRM, LQC).

Since the tea samples contain a very high amount of alkaloids, dilution prior to the chromatographic analysis is mandatory. In this method, a wide dilution factor (1:5000) was used in order to guarantee that the majority of samples will fit the calibration range (1.5 to 400 ng/mL after dilution). The concentration calculated with the linear regression equation was multiplied by 5000 to correct the dilution. One of the samples used for HRM quantification (P1, G1, T1, or C1) could not be quantified by this method once its concentration was above 400 ng/mL. For this reason, HRM stability was evaluated using two samples only.

### 2.2. Long-Term Stability

Although long-term stability had been designed to have equally spaced measurements (every three or four months), frequent instrumental problems allowed only the analysis of time 0 (month 0) and after four and twelve months. For this reason, the regression model used for data analysis could not predict the stability in these samples. However, the model was able to evaluate the influence of time in the concentration variation when samples were kept in the refrigerator (4–8 °C).

**Figure 3:**
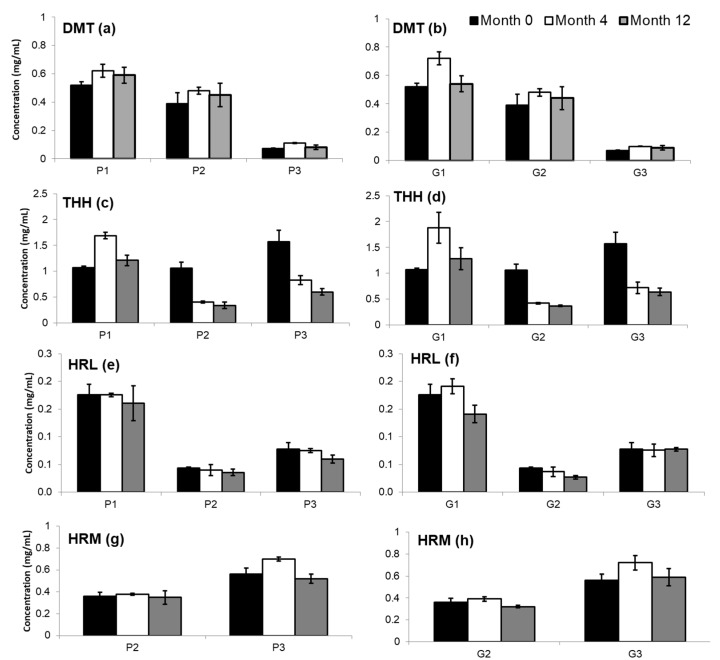
Bars graph (*n* = 3) showing concentration fluctuation over time for (**a**,**b**) DMT, (**c**,**d**) THH, (**e**,**f**) HRL, and (**g**,**h**) HRM in ayahuasca samples stored at 4–8 °C and analyzed at time 0 and after 4 to 12 months either in plastic (P1, P2, and P3) or glass (G1, G2, and G3) containers. Data are presented as mean concentration (mg/mL) ± standard deviations.

HRM and DMT remained stable over the whole period, and no significant variation in their amount was observed over time. Moreover, there were no differences between keeping the samples stored in plastic (P1, P2, and P3) or glass vials (G1, G2, and G3) (Figure 3a,b,g,h). Samples P3 and G2 presented a significant variation in HRL concentration with decreases of 23.7% and 37.9%, respectively (Figure 3e,f). Although most samples reveal no considerable decrease (≤20%), the regression analysis indicates that all samples tend to lose HRL amounts over time (*p* ≤ 0.05). THH, on the other hand, had a remarkable variation in samples P2/G2 and P3/G3, reaching drops as high as 67.9% (Figure 3c,d). In addition, each sample had a different reduction rate over time for this analyte, and there were no differences between keeping the samples in plastic or glass vials.

### 2.3. Transportation Stability

This condition was designed to reproduce the extreme temperatures to which a sample may be exposed during transportation by regular mail in Brazil. As established in previous investigations performed in our laboratory, 37 °C was defined as the average temperature that a sample may be exposed inside the mail boxes. Although ayahuasca tea samples are usually well packed and sent by the faster services available, variation in the tea composition was evaluated for 7 days.

**Figure 4:**
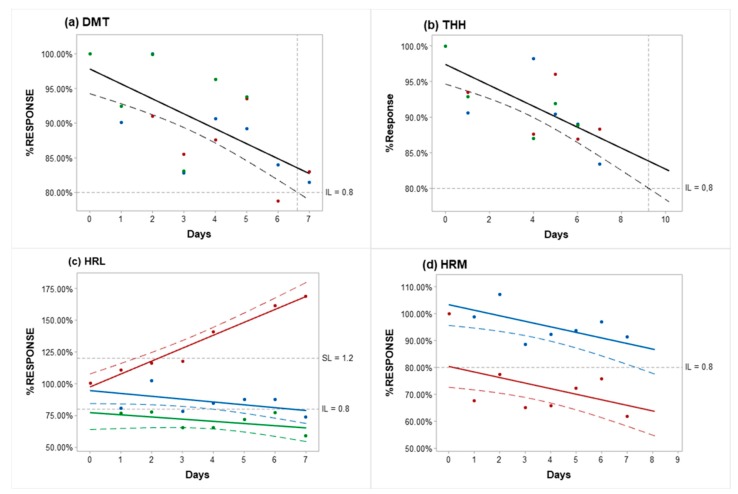
Graphs showing the stability profile over time for (**a**) DMT, (**b**) THH, (**c**) HRL, and (**d**) HRM after seven days of storage at 37 °C. ● Sample T1, ● Sample T2, ● Sample T3. −−− Adjusted line. - - - - IL (inferior limit) or SL (superior limit). Confidence interval = 95%.

In this condition, the DMT and THH profiles were unchanged for all samples (Figure 4a,b); although they tend to diminish over the 7 days of analysis, the decrease was not significant (*p* ≥ 0.05) and did not reach the inferior limit (0.8 or a 20% decrease). The prediction model suggests that DMT concentration is stable for 6.6 days and THH for 9.2 days (r^2^ pred. of 39.7% and 42%, respectively). Each sample reveals a different decrease rate for HRL. In fact, sample T2 reveals unusual behavior with concentration increments exceeding the superior limit after the 3rd day in 40.8% (superior limit equal to 1.2 or a 20% increase). Samples T1 and T3 have a pronounced reduction in HRL concentrations on day 7 (26.5% decrease) and day 1 (23.3%), respectively. Although HRM stability results obtained at 37 °C for 7 days show no considerable decrease in HRM concentration for sample T2, specimen T3 had a different outcome and reached a decline of 32.5% on the 1st day. Despite these differences, both samples were influenced by time, and the HRM concentration shows the tendency to drop at similar rates for both samples (*p* ≤ 0.05).

### 2.4. Freeze-Thaw Cycles

Table 1 shows the percentage of variation for DMT, THH, HRL, and HRM after each cycle for three different samples (C1, C2, and C3). Negative values refer to the loss of analyte concentration, while positive results concern its increments. In general, all analytes were stable after three freeze–thaw cycles, although the regression model reveals a trend in concentration decrease for all samples at a similar rate (*p* ≤ 0.05).

## 3. Discussion 

Beyond the fact that method validation guidelines suggest a stability evaluation whenever this information is not available in the literature [27,28], testing the stability of ayahuasca alkaloids provides valuable information not only for all research groups studying this plant preparation but also for the religious groups consuming the beverage. Those guidelines propose the use of spiked quality control samples at different concentrations kept under the specific conditions expected for real samples [27,28]. Once it is not possible to mimic the whole matrix composition of the tea, given its complex phytochemical profile, this stability evaluation was performed in authentic samples, and the alkaloid variation was calculated by a comparison with time zero responses.

Although previous studies have mentioned stability evaluations of ayahuasca lasting from 80 days to 18 months [16,20,29,30], only one previous study reported long-term (11 months) stability data under refrigerator storage (4–8 °C) [16], a condition usually employed by ayahuasca users and researchers. While this study analyzed only a single batch at two time-points with no statistical data analysis, our investigation comprehensively assessed alkaloid stability in three samples kept in different conditions over several time-points, and all data underwent thorough statistical evaluation, whenever appropriate. Our results demonstrate that DMT and HRM were perfectly stable over 12 months under refrigerator storage, while HRL presented a considerable degradation tendency at different rates for each sample. Moreover, THH concentration suffered huge decreases after a four-month interval in most samples, and each sample had a different decrease profile. There was no difference in keeping samples in plastic or glass vials. Both the previous report [16] and the current study have found DMT and HRM to be stable over time, despite HRM demonstrating a slight tendency to concentration increase (the last time point was 12.6% higher) in the first evaluation. Similarly, in the two investigations, the HRL profile was maintained stable, although a clear trend on concentration decline (19.2% decrease) over the 11 months was observed. Still, while our report indicates that only one out of three THH specimens had no important variation, the previous evaluation showed the single THH sample to be stable at the two time-points.

Samples were kept at 37 °C for 7 days in order to reproduce the possible extreme conditions to which they may be exposed during transportation since ayahuasca preparations are commonly sent by mail to the laboratories responsible for quantifying the material before administration/consumption. After daily analysis, DMT and THH were considered stable over the whole period despite both analytes revealing a trend to decrease. The HRM profile also reveals a tendency to diminish over time, although only the sample with the lower concentration reached the inferior limit on the very first day of analysis. HRL concentrations, on the other hand, showed remarkable fluctuations with both increase and decrease. Inferior and superior limits were exceeded days before the last measurement for the two samples in a way that a variation pattern could not be established.

The concentration variation observed for DMT and the harmala alkaloids in room temperature has been previously reported [20,30]. Despite having found an ayahuasca tea sample to be stable over six months at −20 °C in an amber vial, the authors suggested that a non-linear fluctuation may occur when samples are kept at room temperature and are exposed to light and pH changes due to aging [22]. This fluctuation could be attributed to alkaloid degradation or inter-conversion when samples are exposed to these variables [20,29,30,31]. For instance, the significant alkaloid variation observed in an ayahuasca sample stored at room temperature for 18 months and with variable light exposure (e.g., during dose preparation) could be explained by three factors (temperature, light, pH) [30]. Other sources have suggested that HRL and THH may be formed in situ from HRM reduction or that HRM and THH are formed from HRL through a chemical reduction pathway [20,31]. In any case, we believe that the non-patterned variations found in the β-carbolines concentrations in different conditions are due to a complex process involving alkaloid degradation, inter-conversion, and even leaching from the vegetal material (sludge) usually present on the bottom of the containers used for ayahuasca storage in both religious and scientific contexts.

Finally, according to the *Standard Practices for Method Validation in Forensic Toxicology* guideline, stability must be assessed at three freeze–thaw cycles when freezing the samples prior to analysis is part of the laboratory’s routine [27]. The freeze–thaw stability evaluation is also important in case re-analysis is necessary. This set of experiments demonstrated that DMT, THH, HRL, and HRM remain stable since the concentration decreases were not significant and did not reach 20%.

## 4. Materials and Methods

### 4.1. Standards and Reagents

*N,N*-Dimethyltryptamine (DMT) was purchased from Cerilliant Corporation (Round Rock, TX, USA). Harmine (HRM) and harmaline (HRL) were acquired from Sigma-Aldrich (Saint Louis, MO, USA). The internal standard, deuterated dimethyltryptamine (DMT-*d*_6_), was synthetized as described by [6]. Tetrahydroharmine (THH) was synthetized from harmaline (HRL), according to the method described by [32]. Ammonium formate, formic acid, and methanol HPLC grade were obtained from Merck KGaA (Darmstadt, Germany).

### 4.2. Plant Material and Sample Preparation

Three different ayahuasca tea samples were provided free of charge by a branch of the Santo Daime church (Rainha do Céu) based in Ribeirão Preto-SP, Brazil. Samples were prepared between January 2012 and February 2017 in Ribeirão Preto-SP, Brazil.

Samples were prepared according to a dilute-and-shoot procedure previously validated in our laboratory as proposed by international guidelines [33]. The sample preparation consisted of a dilution procedure using a 2 mM ammonium formate buffer with 0.1% formic acid (solution A) to a final ratio of 1:5000 in three steps (1:10 × 1:10 × 1:50). First, an aliquot of 100 µL was diluted in 900 µL of solution A. Then, 100 uL of the first dilution step was again diluted in 900 µL of solution A. Next, a new aliquot of 100 µL was diluted in 4900 µL of buffer solution A. Finally, 100 uL of this last dilution was added with 10 uL of the internal standard (DMT-*d*_6_ 1 µg/mL). After this procedure, 5 µL of the diluted sample was injected into the LC-ESI-MS/MS system.

### 4.3. Instrumental Analysis

The sample preparation method was fully validated according to international guidelines [32].

Analyses were performed using a Waters UPLC Acquity System coupled to a Quattro Premier tandem MS with electrospray ionization (ESI) operated in the positive ion mode (Waters Corporation, Milford, MA, USA). Chromatographic separation was conducted on a UPLC BEH C18 2.1 mm × 100 mm, ID 1.7 µm Acquity column using the following gradient elution: A (2 mM ammonium formate buffer with 0.1% formic acid) and a mobile phase B (0.1% formic acid in methanol) at a constant flow rate of 300 µL/min; 10%B (0 to 0.5 min); 10%–50%B (0.5 to 7.0 min); 50%–10%B (7.0 to 7.1 min), and 10%B for 8 min. Samples were analyzed using a 5 µL injection volume. The mass spectrometer was operated under the multiple-reaction monitoring mode (MRM), considering three transitions for each analyte. MS settings were established as follows: Desolvation gas flow rate, 1100 L/h; cone gas flow rate, 200 L/h; desolvation temperature, 350 °C; source temperature, 100 °C; capillary voltage, 1000 V. The retention times, capillary voltage, collision energy, and m/z transitions used for quantification of each analyte are indicated in Table 2.

### 4.4. Stability Design and Data Analysis

Stability was evaluated in three different conditions:

(1) Long-term stability: Analyses of three samples every four months for twelve months. Samples were stored at 4–8 °C either in plastic (P1, P2, and P3) or in glass (G1, G2, and G3) containers. Quantification was performed in triplicate for each sample at 0, 4, 8, and 12 months, and the first-month measurements (time zero) were used as controls.

(2) Transportation stability: Designed to mimic the extreme temperatures to which a sample may be exposed during transportation by regular mail in Brazil. The temperature was set at 37 °C, and the three samples T1, T2, and T3 were analyzed daily in triplicate at 0, 1, 2, 3, 4, 5, 6, and 7 days. Day 0 was used as a control.

(3) Freeze–thaw cycles: Performed in three freeze–thaw cycles in which aliquots of C1, C2, and C3 samples were kept at −20 °C for 24 h and thawed to ambient temperature before analysis. Each sample was analyzed in triplicate at time 0 and after 1st, 2nd, and 3rd cycles. Once again, the first quantification, time zero, was used as a control to calculate possible alkaloid degradation.

Microsoft Office Excel^®^ and Minitab^®^ 19 were used for graphics design. The regression and significance analysis were calculated with the stability tool available in the software Minitab^®^ 19. Samples with alkaloid concentration varying between the inferior limit (IL 0.8 or a 20% decrease) and the superior limit (SL 1.2 or a 20% increase) were considered stable over time. The significance of the trends in concentration increase or decrease, as well as the rate in which variation occurs for each sample were also evaluated. Significance was established when a factor showed a *p* value ≤ 0.05 using a confidence interval of 95%.

## 5. Conclusions

The stability evaluation performed in this study provides important information for researchers, ayahuasca users, and analytical laboratory personnel. To our knowledge, this is the most comprehensive evaluation of ayahuasca’s stability profile using statistic tools, rather than a simple comparison between initial and final concentrations usually employed in analytical toxicology. By this approach, it is possible to assess reliable information regarding degradation trends in various matrices and to determine whether the observed variation originates in the analytical measurement or whether it is actually related to analyte loss. We conclude that DMT is stable in all conditions tested (12 months at refrigerator temperature, 7 days at 37 °C, and three freeze–thaw cycles). The harmala alkaloids, by contrast, undergo extensive fluctuation, which is sample-dependent, when exposed to long-term storage and high temperatures. That may occur due to degradation, alkaloid inter-conversion, and even leaching from the bottom vegetal material commonly found in the tea containers. These results suggest that the quantification of the plant preparation is always important before administration or consumption in controlled studies since THH, HRL, and HRM concentrations may vary greatly after being stored for long periods of time and/or high temperatures. Finally, religious users may be able to improve the storage of their entheogenic preparations in order to reach the desirable spiritual outcomes that come from the proper interaction between DMT and harmala alkaloids.

## Figures and Tables

**Table 1:** Percentage of variation for DMT, THH, HRL, and HRM after each of the three freeze–thaw cycles.

Sample	Cycle	DMT%	THH%	HRL%	HRM%
C1	1st	−3.9	3.8	−17.4	*
	2nd	1.3	7.7	−6.1	*
	3rd	−8.4	−19.0	−13.6	*
C2	1st	−6.0	3.7	4.4	4.6
	2nd	−3.5	6.5	3.6	7.9
	3rd	−16.3	−13.5	−14.2	−9.9
C3	1st	−1.2	−7.1	1.7	−9.0
	2nd	1.0	18.2	8.4	16.1
	3rd	−14.1	−18.8	−13.6	−10.6

* Data not available. HRM concentration was over the calibration range.

**Table 2:** Precursor ions, product ions, retention times, cone voltage, and collision energy for all analytes.

Analyte	Retention Time (min.)	Precursor Ion (*m*/*z*)	Product Ion (*m*/*z*)	Cone Voltage	Collision Energy
DMT-*d*_6_ (IS)	2.87	195.1	63.9 ^1^	15	14
			114.9	15	36
			143.8	15	22
DMT	2.88	188.9	57.8 ^1^	25	11
			116.7	25	29
			143.8	25	17
THH	4.37	217.1	172.8	25	29
			187.9 ^1^	25	17
			200.0	25	13
HRL	5.27	215.2	130.4	50	41
			171.7 ^1^	50	33
			199.9	50	25
HRM	5.56	213.2	143.8	50	41
			169.8 ^1^	50	33
			198.0	50	25

^1^ Transition used for analyte quantification. IS—internal standard.
